# Hexa­aqua­dodeca-μ_2_-iodido-*octahedro*-hexa­tantalum diiodide tetra­hydrate

**DOI:** 10.1107/S2414314621003047

**Published:** 2021-03-31

**Authors:** Florian Schröder, Martin Köckerling

**Affiliations:** a Universität Rostock, Institut für Chemie, Anorganische Festkörperchemie, Albert-Einstein-Str. 3a, D-18059 Rostock, Germany; Vienna University of Technology, Austria

**Keywords:** crystal structure, metal cluster, tantalum, iodide

## Abstract

In the crystal structure of the cluster salt, [Ta_6_I_12_(H_2_O)_6_]I_2_·4H_2_O, the octa­hedral {Ta_6_} cluster core is μ_2-_coordinated by twelve iodido ligands (inner ligand sphere) whereas the six aqua ligands coordinate each at the six outer positions. The discrete, inversion-symmetric cluster complex is double-positively charged, and two iodide anions are present in the crystal structure as counter-ions. In addition, four water mol­ecules are co-crystallized. Hydrogen bonds between the cluster unit, the iodide anions and co-crystallized water mol­ecules stabilize the charge-assisted packing in the crystal structure.

## Abstract 2

The crystal structure of the title compound comprises a [Ta_6_I_12_(H_2_O)_6_]^2+^ unit, two I^−^ counter-anions and four water mol­ecules of crystallization. The charge-assisted crystal packing is consolidated by hydrogen bonds.

## Graphical abstract

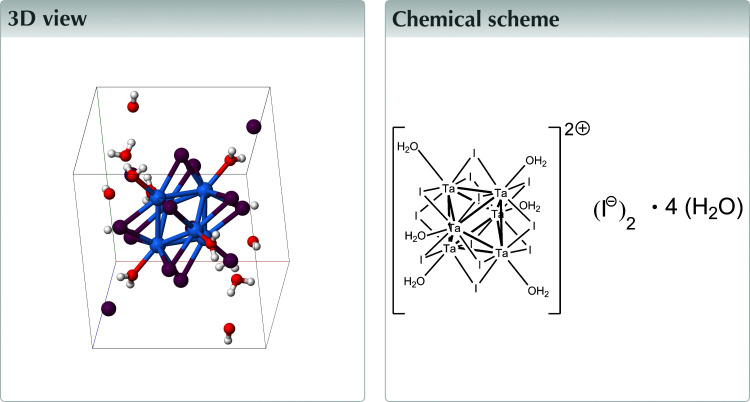

## Structure description

Cluster complexes with strong metal-metal bonds have been in the focus of research activities for a long time (Cotton, 1964 ▸; Simon, 1988 ▸). Starting from the well-known compound [Ta_6_I_14_] (Bauer *et al.*, 1965 ▸), the title compound [Ta_6_I_12_(H_2_O)_6_]I_2_·4H_2_O was obtained by reaction with a water–acetone mixture and subsequent evaporation of the solvent. This compound was previously mentioned by Schäfer *et al.* (1972 ▸) and Shamshurin *et al.* (2019 ▸), however, without determination of its crystal structure. [Ta_6_I_12_(H_2_O)_6_]I_2_·4H_2_O can be used efficiently as a precursor for new tantalum cluster compounds.

**Figure 1:**
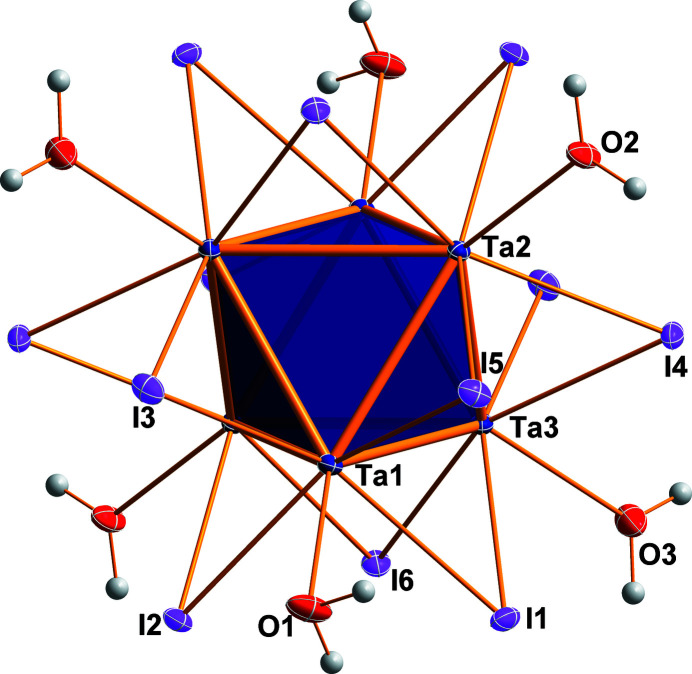
The centrosymmetric cluster cation [Ta_6_I_12_(H_2_O)_6_]^2+^ in the crystal structure of [Ta_6_I_12_(H_2_O)_6_]I_2_·4H_2_O with the atoms shown as displacement ellipsoids at the 50% probability level. Non-labelled atoms are generated by the symmetry operation −*x* + 1, −*y* + 1, −*z* + 1.

**Figure 2:**
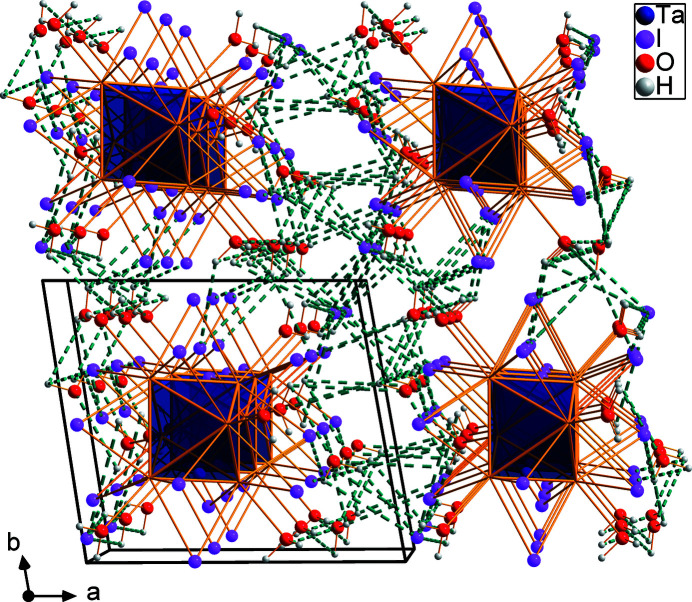
Packing of cluster units, iodide anions, and co-crystallized water mol­ecules of [Ta_6_I_12_(H_2_O)_6_]I_2_·4H_2_O in a view along the *c* axis with O—H⋯O and O—H⋯I hydrogen bonds shown as blue–green dashed lines.

The metal atoms of the {Ta_6_} unit are octa­hedrally arranged (point group symmetry 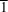 ), with an average Ta—Ta bond length of 2.934 Å. The twelve μ_2_-bridging positions of the inner ligand sphere are occupied by iodido ligands (Fig. 1 ▸). The average Ta—I bond length is 2.809 Å and the average Ta—I—Ta angle is 63.1°. The six positions of the outer ligand sphere are occupied by aqua ligands (O1, O2, and O3). The average Ta—O bond length is 2.286 Å. All inter­atomic distances and angles within the cluster complex match well with those in comparable compounds of the same charge (Shamshurin *et al.*, 2019 ▸). Based on the anion:cation ratio and the bond lengths, 16 cluster-based electrons (CBE) are present. The double-positive charge of the cluster cation is counter-balanced by two iodide ions (I7). Two water mol­ecules (O4, O5) are co-crystallized per asymmetric unit, which are connected to the cluster complex *via* H⋯I and H⋯O hydrogen bonds. Further hydrogen bonds exist between some of the ligating I atoms, the iodide counter-anions and water mol­ecules. Numerical details of the hydrogen-bonding inter­actions up to *D*⋯*A* distances of 3.7 Å are given in Table 1 ▸. A packing plot with a view along the crystallographic *c* direction is displayed in Fig. 2 ▸.

## Synthesis and crystallization

Under Schlenk conditions the starting material, *viz*. the cluster compound [Ta_6_I_14_], was produced analogously to a literature procedure (Bauer *et al.*, 1965 ▸) and subsequently finely ground under protective gas by means of a ball mill. The obtained powder was pyrophoric in air. 400 mg (139.75 µmol) of [Ta_6_I_14_] were stirred under argon in an intensely degassed solution of 30 ml (1.67 mol) water and 30 ml (0.40 mol) acetone at room temperature for one day. After filtration, an intense green solution was obtained. The solvent was slowly evaporated in air at room temperature. After several days, black single crystals had formed, which were washed several times with water. 180 mg (59.16 µmol, yield: 45%) of [Ta_6_I_12_(H_2_O)_6_]I_2_·4H_2_O were obtained. NMR, IR and elemental analysis confirmed the composition determined by the X-ray structural analysis. Details of the complementary analytical methods are given in the supplementary information.

## Refinement

Crystal data, data collection and structure refinement details are summarized in Table 2 ▸. Hydrogen atoms were placed on idealized positions and refined using a riding model with *U*_iso_(H) = 1.5*U*_eq_(O).

## Supplementary Material

Crystal structure: contains datablock(s) I. DOI: 10.1107/S2414314621003047/wm4145sup1.cif

Structure factors: contains datablock(s) I. DOI: 10.1107/S2414314621003047/wm4145Isup2.hkl

Supporting Information. DOI: 10.1107/S2414314621003047/wm4145sup3.pdf

CCDC reference: 2072508

Additional supporting information:  crystallographic information; 3D view; checkCIF report

## Figures and Tables

**Table 1:** Hydrogen-bond geometry (Å, °)

*D*—H⋯*A*	*D*—H	H⋯*A*	*D*⋯*A*	*D*—H⋯*A*
O1—H1*A*⋯I1	0.85	2.71	3.196 (5)	118
O1—H1*B*⋯I5	0.85	2.66	3.226 (4)	126
O2—H2*B*⋯O5	0.85	1.85	2.618 (6)	150
O2—H2*A*⋯I7^i^	0.85	2.70	3.534 (4)	169
O3—H3*B*⋯I7^ii^	0.85	2.80	3.455 (4)	135
O3—H3*A*⋯I7^iii^	0.85	2.75	3.488 (4)	146
O4—H4*A*⋯O1	0.85	2.16	2.685 (7)	120
O4—H4*B*⋯I6^iv^	0.85	2.95	3.695 (5)	148
O5—H5*B*⋯O4^v^	0.85	2.09	2.894 (8)	157
O5—H5*A*⋯I7^vi^	0.85	2.82	3.563 (5)	147

Symmetry codes: (i) 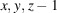 ; (ii) 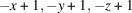 ; (iii) 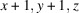 ; (iv) 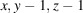 ; (v) 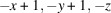 ; (vi) 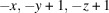 .

**Table 2:** Experimental details

Crystal data	
Chemical formula	[Ta_6_I_12_(H_2_O)_6_]I_2_·4H_2_O
*M*_r_	3042.46
Crystal system, space group	Triclinic, *P* 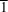
Temperature (K)	123
*a*, *b*, *c* (Å)	10.009 (2), 10.118 (2), 10.498 (2)
α, β, γ (°)	117.451 (4), 97.465 (4), 96.344 (4)
*V* (Å^3^)	917.7 (2)
*Z*	1
Radiation type	Mo *K*α
μ (mm^−1^)	29.32
Crystal size (mm)	0.04 × 0.03 × 0.03

Data collection	
Diffractometer	Bruker APEXII CCD
Absorption correction	Multi-scan (*SADABS*; Krause *et al.*, 2015 ▸)
No. of measured, independent and observed [*I* > 2σ(*I*)] reflections	39077, 5847, 5081
*R*_int_	0.057
(sin θ/λ)_max_ (Å^−1^)	0.724

Refinement	
*R*[*F* ^2^ > 2σ(*F* ^2^)], *wR*(*F* ^2^), *S*	0.025, 0.051, 1.07
No. of reflections	5847
No. of parameters	136
H-atom treatment	H-atom parameters constrained
Δρ_max_, Δρ_min_ (e Å^−3^)	1.93, −1.52

Computer programs: *APEX2* (Bruker, 2017 ▸), *SAINT* (Bruker, 2017 ▸), *SHELXT2014* (Sheldrick, 2015*a* ▸), *SHELXL2014* (Sheldrick, 2015*b* ▸), *DIAMOND* (Brandenburg & Putz, 2019 ▸).

## full crystallographic data

### Crystal data

**Table:**

[Ta_6_I_12_(H_2_O)_6_]I_2_·4H_2_O	*Z* = 1
*M**_r_* = 3042.46	*F*(000) = 1280
Triclinic, *P*1	*D*_x_ = 5.505 Mg m^−^^3^
*a* = 10.009 (2) Å	Mo *K*α radiation, λ = 0.71073 Å
*b* = 10.118 (2) Å	Cell parameters from 9987 reflections
*c* = 10.498 (2) Å	θ = 2.3–32.8°
α = 117.451 (4)°	µ = 29.32 mm^−^^1^
β = 97.465 (4)°	*T* = 123 K
γ = 96.344 (4)°	Block, black
*V* = 917.7 (2) Å^3^	0.04 × 0.03 × 0.03 mm

### Data collection

**Table:**

Bruker APEXII CCD diffractometer	5081 reflections with *I* > 2σ(*I*)
Radiation source: microfocus sealed tube	*R*_int_ = 0.057
φ and ω scans	θ_max_ = 31.0°, θ_min_ = 3.1°
Absorption correction: multi-scan (SADABS; Krause *et al.*, 2015)	*h* = −14→14
	*k* = −14→14
39077 measured reflections	*l* = −15→15
5847 independent reflections	

### Refinement

**Table:**

Refinement on *F*^2^	Primary atom site location: structure-invariant direct methods
Least-squares matrix: full	Secondary atom site location: difference Fourier map
*R*[*F*^2^ > 2σ(*F*^2^)] = 0.025	Hydrogen site location: difference Fourier map
*wR*(*F*^2^) = 0.051	H-atom parameters constrained
*S* = 1.07	*w* = 1/[σ^2^(*F*_o_^2^) + (0.0139*P*)^2^ + 1.4885*P*] where *P* = (*F*_o_^2^ + 2*F*_c_^2^)/3
5847 reflections	(Δ/σ)_max_ = 0.001
136 parameters	Δρ_max_ = 1.93 e Å^−^^3^
0 restraints	Δρ_min_ = −1.52 e Å^−^^3^

### Special details

**Table:**

Geometry. All esds (except the esd in the dihedral angle between two l.s. planes) are estimated using the full covariance matrix. The cell esds are taken into account individually in the estimation of esds in distances, angles and torsion angles; correlations between esds in cell parameters are only used when they are defined by crystal symmetry. An approximate (isotropic) treatment of cell esds is used for estimating esds involving l.s. planes.
Refinement. Hydrogen atoms were placed on idealized positions and refined using a riding model with *U*_iso_(H) = 1.5*U*_eq_(O).

### Fractional atomic coordinates and isotropic or equivalent isotropic displacement parameters (Å^2^)

**Table:**

	*x*	*y*	*z*	*U*_iso_*/*U*_eq_	
Ta1	0.59756 (2)	0.32569 (3)	0.37992 (2)	0.00826 (5)	
Ta2	0.38779 (2)	0.47496 (3)	0.30732 (2)	0.00757 (5)	
Ta3	0.65272 (2)	0.65637 (3)	0.49926 (2)	0.00823 (5)	
I1	0.42859 (4)	0.06430 (4)	0.34481 (4)	0.01322 (8)	
I2	0.47893 (4)	0.23469 (4)	0.08727 (4)	0.01115 (7)	
I3	0.82943 (4)	0.47721 (4)	0.34401 (4)	0.01372 (8)	
I4	0.55445 (4)	0.66827 (4)	0.24333 (4)	0.01211 (7)	
I5	0.77566 (4)	0.30105 (4)	0.59483 (4)	0.01271 (8)	
I6	0.85016 (4)	0.73689 (4)	0.75183 (4)	0.01137 (7)	
I7	0.07640 (4)	0.11686 (4)	0.77730 (4)	0.01530 (8)	
O1	0.7026 (5)	0.1315 (5)	0.2412 (5)	0.0188 (9)	
H1A	0.6296	0.0645	0.1957	0.028*	
H1B	0.7414	0.1157	0.3085	0.028*	
O2	0.2672 (4)	0.4452 (5)	0.0938 (4)	0.0153 (9)	
H2A	0.2195	0.3610	0.0260	0.023*	
H2B	0.2511	0.5200	0.0820	0.023*	
O3	0.8193 (5)	0.8295 (5)	0.4994 (5)	0.022 (1)	
H3A	0.8500	0.9175	0.5723	0.032*	
H3B	0.8760	0.8111	0.4425	0.032*	
O4	0.8053 (5)	0.1201 (6)	0.0131 (6)	0.031 (1)	
H4A	0.8386	0.1350	0.0984	0.047*	
H4B	0.7796	0.0248	−0.0424	0.047*	
O5	0.1291 (6)	0.6203 (6)	0.0313 (6)	0.037 (1)	
H5A	0.0548	0.6462	0.0566	0.055*	
H5B	0.1667	0.6835	0.0082	0.055*	

### Atomic displacement parameters (Å^2^)

**Table:**

	*U*^11^	*U*^22^	*U*^33^	*U*^12^	*U*^13^	*U*^23^
Ta1	0.0091 (1)	0.0076 (1)	0.0067 (1)	0.00218 (8)	0.00069 (8)	0.00236 (9)
Ta2	0.0082 (1)	0.0071 (1)	0.0065 (1)	0.00093 (8)	0.00006 (8)	0.00295 (9)
Ta3	0.0086 (1)	0.0078 (1)	0.0069 (1)	−0.00055 (8)	0.00034 (8)	0.00315 (9)
I1	0.0187 (2)	0.0072 (2)	0.0119 (2)	0.0013 (1)	0.0001 (1)	0.0041 (1)
I2	0.0136 (2)	0.0110 (2)	0.0069 (2)	0.0035 (1)	0.0012 (1)	0.0027 (1)
I3	0.0095 (2)	0.0174 (2)	0.0116 (2)	0.0018 (1)	0.0037 (1)	0.0047 (2)
I4	0.0149 (2)	0.0117 (2)	0.0096 (2)	−0.0014 (1)	0.0003 (1)	0.0064 (2)
I5	0.0136 (2)	0.0130 (2)	0.0104 (2)	0.0060 (1)	0.0002 (1)	0.0045 (2)
I6	0.0088 (2)	0.0122 (2)	0.0102 (2)	−0.0013 (1)	−0.0010 (1)	0.0045 (2)
I7	0.0144 (2)	0.0138 (2)	0.0140 (2)	−0.0006 (2)	0.0002 (1)	0.0051 (2)
O1	0.021 (2)	0.015 (2)	0.014 (2)	0.010 (2)	0.001 (2)	0.001 (2)
O2	0.021 (2)	0.013 (2)	0.009 (2)	0.005 (2)	−0.002 (2)	0.004 (2)
O3	0.021 (2)	0.022 (2)	0.014 (2)	−0.010 (2)	0.002 (2)	0.006 (2)
O4	0.031 (3)	0.028 (3)	0.021 (3)	0.005 (2)	0.006 (2)	0.002 (2)
O5	0.057 (4)	0.042 (3)	0.029 (3)	0.032 (3)	0.017 (3)	0.025 (3)

### Geometric parameters (Å, º)

**Table:**

Ta1—O1	2.303 (4)	Ta3—I4	2.7996 (6)
Ta1—I3	2.8022 (5)	Ta3—I6	2.8090 (6)
Ta1—I2	2.8104 (6)	Ta3—I1^i^	2.8141 (6)
Ta1—I5	2.8110 (5)	Ta3—Ta1^i^	2.9380 (5)
Ta1—I1	2.8181 (6)	Ta3—Ta2^i^	2.9386 (5)
Ta1—Ta2^i^	2.9281 (5)	I1—Ta3^i^	2.8140 (6)
Ta1—Ta3	2.9352 (6)	I5—Ta2^i^	2.8245 (5)
Ta1—Ta2	2.9354 (4)	I6—Ta2^i^	2.8131 (5)
Ta1—Ta3^i^	2.9379 (5)	O1—H1A	0.8500
Ta2—O2	2.273 (4)	O1—H1B	0.8501
Ta2—I4	2.8001 (5)	O2—H2A	0.8500
Ta2—I6^i^	2.8131 (5)	O2—H2B	0.8500
Ta2—I2	2.8175 (5)	O3—H3A	0.8500
Ta2—I5^i^	2.8245 (5)	O3—H3B	0.8501
Ta2—Ta1^i^	2.9281 (5)	O4—H4A	0.8501
Ta2—Ta3	2.9303 (5)	O4—H4B	0.8499
Ta2—Ta3^i^	2.9386 (5)	O5—H5A	0.8501
Ta3—O3	2.281 (4)	O5—H5B	0.8500
Ta3—I3	2.7936 (5)		
			
O1—Ta1—I3	76.7 (1)	Ta3—Ta2—Ta1	60.05 (1)
O1—Ta1—I2	75.0 (1)	O2—Ta2—Ta3^i^	135.2 (1)
I3—Ta1—I2	87.76 (2)	I4—Ta2—Ta3^i^	148.61 (1)
O1—Ta1—I5	77.5 (1)	I6^i^—Ta2—Ta3^i^	58.42 (1)
I3—Ta1—I5	86.83 (2)	I2—Ta2—Ta3^i^	99.08 (2)
I2—Ta1—I5	152.47 (1)	I5^i^—Ta2—Ta3^i^	99.76 (1)
O1—Ta1—I1	76.5 (1)	Ta1^i^—Ta2—Ta3^i^	60.04 (1)
I3—Ta1—I1	153.17 (1)	Ta3—Ta2—Ta3^i^	90.19 (1)
I2—Ta1—I1	86.94 (1)	Ta1—Ta2—Ta3^i^	60.02 (1)
I5—Ta1—I1	85.83 (2)	O3—Ta3—I3	77.0 (1)
O1—Ta1—Ta2^i^	136.4 (1)	O3—Ta3—I4	76.5 (1)
I3—Ta1—Ta2^i^	99.12 (1)	I3—Ta3—I4	86.67 (2)
I2—Ta1—Ta2^i^	148.59 (1)	O3—Ta3—I6	76.7 (1)
I5—Ta1—Ta2^i^	58.92 (1)	I3—Ta3—I6	85.83 (2)
I1—Ta1—Ta2^i^	99.05 (1)	I4—Ta3—I6	153.15 (2)
O1—Ta1—Ta3	134.9 (1)	O3—Ta3—I1^i^	76.0 (1)
I3—Ta1—Ta3	58.22 (1)	I3—Ta3—I1^i^	152.98 (2)
I2—Ta1—Ta3	99.81 (1)	I4—Ta3—I1^i^	87.12 (2)
I5—Ta1—Ta3	100.16 (1)	I6—Ta3—I1^i^	87.96 (2)
I1—Ta1—Ta3	148.59 (1)	O3—Ta3—Ta2	135.0 (1)
Ta2^i^—Ta1—Ta3	60.156 (9)	I3—Ta3—Ta2	99.94 (2)
O1—Ta1—Ta2	133.6 (1)	I4—Ta3—Ta2	58.46 (1)
I3—Ta1—Ta2	99.61 (2)	I6—Ta3—Ta2	148.36 (1)
I2—Ta1—Ta2	58.68 (1)	I1^i^—Ta3—Ta2	99.10 (2)
I5—Ta1—Ta2	148.84 (1)	O3—Ta3—Ta1	135.5 (1)
I1—Ta1—Ta2	99.98 (2)	I3—Ta3—Ta1	58.51 (1)
Ta2^i^—Ta1—Ta2	89.92 (1)	I4—Ta3—Ta1	98.94 (1)
Ta3—Ta1—Ta2	59.89 (1)	I6—Ta3—Ta1	98.98 (1)
O1—Ta1—Ta3^i^	134.9 (1)	I1^i^—Ta3—Ta1	148.52 (1)
I3—Ta1—Ta3^i^	148.32 (1)	Ta2—Ta3—Ta1	60.059 (9)
I2—Ta1—Ta3^i^	99.26 (2)	O3—Ta3—Ta1^i^	134.6 (1)
I5—Ta1—Ta3^i^	99.46 (2)	I3—Ta3—Ta1^i^	148.40 (1)
I1—Ta1—Ta3^i^	58.49 (1)	I4—Ta3—Ta1^i^	99.99 (2)
Ta2^i^—Ta1—Ta3^i^	59.94 (1)	I6—Ta3—Ta1^i^	99.87 (2)
Ta3—Ta1—Ta3^i^	90.11 (1)	I1^i^—Ta3—Ta1^i^	58.63 (1)
Ta2—Ta1—Ta3^i^	60.04 (1)	Ta2—Ta3—Ta1^i^	59.86 (1)
O2—Ta2—I4	76.1 (1)	Ta1—Ta3—Ta1^i^	89.89 (1)
O2—Ta2—I6^i^	76.8 (1)	O3—Ta3—Ta2^i^	135.2 (1)
I4—Ta2—I6^i^	152.96 (1)	I3—Ta3—Ta2^i^	99.07 (1)
O2—Ta2—I2	75.7 (1)	I4—Ta3—Ta2^i^	148.25 (1)
I4—Ta2—I2	86.51 (2)	I6—Ta3—Ta2^i^	58.56 (1)
I6^i^—Ta2—I2	86.90 (2)	I1^i^—Ta3—Ta2^i^	100.00 (2)
O2—Ta2—I5^i^	77.3 (1)	Ta2—Ta3—Ta2^i^	89.81 (1)
I4—Ta2—I5^i^	87.40 (2)	Ta1—Ta3—Ta2^i^	59.80 (1)
I6^i^—Ta2—I5^i^	86.68 (2)	Ta1^i^—Ta3—Ta2^i^	59.935 (9)
I2—Ta2—I5^i^	153.00 (1)	Ta3^i^—I1—Ta1	62.88 (1)
O2—Ta2—Ta1^i^	135.8 (1)	Ta1—I2—Ta2	62.88 (1)
I4—Ta2—Ta1^i^	100.22 (2)	Ta3—I3—Ta1	63.28 (1)
I6^i^—Ta2—Ta1^i^	99.05 (1)	Ta3—I4—Ta2	63.11 (1)
I2—Ta2—Ta1^i^	148.52 (1)	Ta1—I5—Ta2^i^	62.61 (1)
I5^i^—Ta2—Ta1^i^	58.47 (1)	Ta3—I6—Ta2^i^	63.03 (1)
O2—Ta2—Ta3	134.6 (1)	Ta1—O1—H1A	96.4
I4—Ta2—Ta3	58.44 (1)	Ta1—O1—H1B	99.0
I6^i^—Ta2—Ta3	148.60 (1)	H1A—O1—H1B	107.7
I2—Ta2—Ta3	99.77 (2)	Ta2—O2—H2A	123.6
I5^i^—Ta2—Ta3	99.33 (2)	Ta2—O2—H2B	122.5
Ta1^i^—Ta2—Ta3	60.20 (1)	H2A—O2—H2B	112.6
O2—Ta2—Ta1	134.1 (1)	Ta3—O3—H3A	123.3
I4—Ta2—Ta1	98.93 (2)	Ta3—O3—H3B	126.6
I6^i^—Ta2—Ta1	99.84 (2)	H3A—O3—H3B	107.7
I2—Ta2—Ta1	58.44 (1)	H4A—O4—H4B	107.7
I5^i^—Ta2—Ta1	148.55 (1)	H5A—O5—H5B	107.7
Ta1^i^—Ta2—Ta1	90.08 (1)		

Symmetry code: (i) −*x*+1, −*y*+1, −*z*+1.

### Hydrogen-bond geometry (Å, º)

**Table:**

*D*—H···*A*	*D*—H	H···*A*	*D*···*A*	*D*—H···*A*
O1—H1*A*···I1	0.85	2.71	3.196 (5)	118
O1—H1*B*···I5	0.85	2.66	3.226 (4)	126
O2—H2*B*···O5	0.85	1.85	2.618 (6)	150
O2—H2*A*···I7^ii^	0.85	2.70	3.534 (4)	169
O3—H3*B*···I7^i^	0.85	2.80	3.455 (4)	135
O3—H3*A*···I7^iii^	0.85	2.75	3.488 (4)	146
O4—H4*A*···O1	0.85	2.16	2.685 (7)	120
O4—H4*B*···I6^iv^	0.85	2.95	3.695 (5)	148
O5—H5*B*···O4^v^	0.85	2.09	2.894 (8)	157
O5—H5*A*···I7^vi^	0.85	2.82	3.563 (5)	147
O4—H4*A*···I3	0.85	3.25	3.633 (5)	111
O4—H4*B*···I1^vii^	0.85	3.23	3.656 (5)	113
O4—H4*A*···I6^viii^	0.85	3.13	3.670 (5)	124
O5—H5*B*···I3^v^	0.85	3.29	3.688 (5)	112
O1—H1*A*···I2^vii^	0.85	3.05	3.745 (4)	140
O4—H4*B*···I2^vii^	0.85	3.29	3.945 (5)	135

Symmetry codes: (i) −*x*+1, −*y*+1, −*z*+1; (ii) *x*, *y*, *z*−1; (iii) *x*+1, *y*+1, *z*; (iv) *x*, *y*−1, *z*−1; (v) −*x*+1, −*y*+1, −*z*; (vi) −*x*, −*y*+1, −*z*+1; (vii) −*x*+1, −*y*, −*z*; (viii) −*x*+2, −*y*+1, −*z*+1.
