# Supplementary material for: Hexa­aqua­dodeca-μ2-iodido-octahedro-hexa­tantalum diiodide tetra­hydrate
Source: IUCrdata. 2021 Mar 31;6(Pt 3):x210304. doi: 10.1107/S2414314621003047 (PMC9462323; doi:10.1107/S2414314621003047)
Supplement: Supplementary file 3 [file x-06-x210304-sup3.pdf]

IUCRData - Supporting information

$Ta_6I_{12}(H_2O)_6]I_2·4H_2O$

The crystals obtained are washed several times with water and dried for six hours in high vacuum (10^{-6} mbar) at 60 °C. The co-crystallized water molecules are thereby removed from the cluster material.

Elemental analysis: M [$H_{12}I_{14}O_6Ta_6$] = 2970.379 g·mol^{-1}: found H = 0.42 % (calc. 0.41 %).

NMR: MeCN-d_3 is dried by refluxed for several hours with CaH_2 and finally distilled under Schlenk conditions.

^{1}H NMR (MeCN-d_3), 300 MHz, 300 K, ppm): δ = 2.13 (s, 12H, H_2O).

IR (300 K, ATR, cm^{-1}): ṽ = 577 (s), 1581 (m), 1628 (m), 2108 (w), 2353 (w), 3204 (w).
